# Altered Gene Response to Aflatoxin B_1_ in the Spleens of Susceptible and Resistant Turkeys

**DOI:** 10.3390/toxins11050242

**Published:** 2019-04-28

**Authors:** Kent M. Reed, Kristelle M. Mendoza, Roger A. Coulombe

**Affiliations:** 1 Department of Veterinary and Biomedical Sciences, College of Veterinary Medicine, University of Minnesota, Saint Paul, MN 55108, USA; mendo008@umn.edu; 2 Department of Animal, Dairy and Veterinary Sciences, College of Agriculture, Utah State University, Logan, UT 84322, USA; roger@usu.edu

**Keywords:** Poultry, turkey, transcriptome, aflatoxin B_1_, spleen, RNAseq

## Abstract

Susceptibility and/or resistance to aflatoxin B_1_ (AFB_1_) is a threshold trait governed principally by glutathione S transferase (GST)-mediated detoxification. In poultry, domesticated turkeys are highly sensitive to AFB_1_, most likely due to dysfunction in hepatic GSTs. In contrast, wild turkeys are comparatively resistant to aflatoxicosis due to the presence of functional hepatic GSTAs and other possible physiological and immunological interactions. The underlying genetic basis for the disparate GST function in turkeys is unknown as are the broader molecular interactions that control the systemic response. This study quantifies the effects of dietary AFB_1_ on gene expression in the turkey spleen, specifically contrasting genetically distinct domesticated (DT, susceptible) and Eastern wild (EW, resistant) birds. Male turkey poults were subjected to a short-term AFB_1_ treatment protocol with feed supplemented with 320 ppb AFB_1_ beginning on day 15 of age and continuing for 14 days. Spleen tissues were harvested and subjected to deep RNA sequencing and transcriptome analysis. Analysis of differential gene expression found the effects of AFB_1_ treatment on the spleen transcriptomes considerably more prominent in the DT birds compared to EW. However, expression of the differentially expressed genes (DEGs) was directionally biased, with the majority showing higher expression in EW (i.e., down-regulation in DT). Significantly altered pathways included FXR/RXR and LXR/RXR activation, coagulation system, prothrombin activation, acute phase response, and atherosclerosis signaling. Differential extra-hepatic expression of acute phase protein genes was confirmed by quantitative real time PCR (qRT-PCR) in the original experiment and additional turkey lines. Results demonstrate that wild turkeys possess a capacity to more effectively respond to AFB_1_ exposure.

## 1. Introduction

Aflatoxins are mycotoxins produced by a group of heterologous fungal strains of *Aspergillus*. Of these mycotoxins, aflatoxin B_1_ (AFB_1_) is the most hepatotoxic, mutagenic, and prevalent worldwide. It is ubiquitous in foods and feeds, and poses a significant health risk to people and animals. Rapidly absorbed in the small intestine, AFB_1_ is metabolized primarily in the liver where it is bioactivated by hepatic cytochrome P450 (CYP) enzymes into the reactive and electrophilic exo-AFB1-8,9-epoxide (AFBO). This reactive epoxide can initiate toxicity by binding to DNA, RNA, proteins, and other critical cellular macromolecules. The principal route of AFB_1_ detoxification is through glutathione S-transferase (GST) enzymes that conjugate AFBO with glutathione (GSH). The principal determinant of species sensitivity to AFB_1_ is the rate and efficiency of GST activity [1].

Poultry are among the most vulnerable animals to AFB_1_ displaying a wide array of adverse effects including reduced feed intake, weight gain, and increased mortality, hepatotoxicity, and GI hemorrhaging [1,2,3,4,5]. Due to likely dysfunction in hepatic GSTs rendering them unable to detoxify AFB_1_, domesticated turkeys (DT) are highly sensitive to AFB_1_ [6,7,8,9,10]. Studies in turkey [11,12] show significant association between AFB_1_ exposure and expression of hepatic phase I and phase II metabolism genes and other genes important in cellular regulation, modulation of apoptosis, and inflammatory responses.

In addition to its hepatotoxic and mutagenic effects, AFB_1_ is a potent immunotoxin acting to suppress cell-mediated, humoral and phagocytic functions [13,14,15]. In susceptible species, the mechanisms of AFB_1_ immunotoxic effects vary depending on the level and duration of exposure. Acute exposure tends to result in immunosuppressive effects, whereas chronic exposure typically produces an inflammatory response and apoptosis [16,17]. Immunosuppressive effects may result from inhibition of antibody production and lymphocyte proliferation by blocked protein synthesis [18] or apoptosis in splenocytes resulting from oxidative stress and DNA damage [17,19]. Key inflammatory responses include monocyte infiltration in the liver [3,20] and expression of pro-inflammatory cytokines [21,22]. In domesticated turkeys, short-term AFB_1_ exposure suppressed transcripts of innate immune genes in the spleen, especially antimicrobial genes. Conversely, transcripts from the protein ubiquitination pathway and multiple interleukin-2 response genes were increased suggesting increased cytotoxic potential or activation-induced cell death during aflatoxicosis [23]. As a consequence of AFB_1_-caused immunosuppression, exposed poultry have lower resistance to secondary infections and diseases [5,24].

By contrast to modern domesticated turkeys, wild turkeys are relatively resistant to aflatoxicosis [25], most likely due, in large part, to the expression and presence of functional hepatic GSTAs [10]. As part of this genetic effect, we found constitutive expression of hepatic *GSTA3* is significantly higher in wild compared to domesticated turkeys [12]. Expression was also significantly higher in AFB_1_-treated birds compared to controls. In the intestine, AFB_1_ significantly up-regulated expression of the primary AFB_1_-activating P450 (*CYP1A5*) and produced transcriptional changes in several tight junction proteins [26]. Significant down-regulation was seen in domesticated birds for numerous pro-inflammatory cytokines, *TGF-β* and *EGF* and gene pathway analysis suggested AFB_1_ suppression of enteroendocrine cells. We hypothesized that as in the liver and intestine, the transcriptome of the spleen would be significantly affected by AFB_1_ and response in turkey would vary by genetic type (wild vs. domestic). This study was designed to quantify the effects of dietary AFB_1_ on gene expression in the turkey spleen, specifically contrasting genetically distinct domesticated (DT, susceptible) and Eastern wild (EW, resistant) birds.

## 2. Results

Sequencing of RNA libraries produced an average of 12.7 M reads per library (range 8.4 M to 15.7 M per individual, Table S1). After trimming and filtering, median Q scores among the forward and reverse reads were consistently high and ranged from 36.4 to 37.2. Average reads per treatment group ranged from 9.6 M to 13.8 M. Of the quality-trimmed reads, ~90% mapped to the annotated turkey gene set (NCBI Annotation 101, Table S1). Based on map position, the estimated mean insert of the libraries was 187.2 bp.

### 2.1. Splenic Gene Expression

Evidence of expression (mapped reads >1.0) was detected for 19,564 genes (mean read depth = 375.4, Table S2). The number of expressed genes per individual averaged 17,406 (Table S1) representing 82.8% of the turkey gene set (tRNAs excluded). When limited to genes with mapped reads ≥3.0 (normalized), the number of expressed genes ranged from 15,886 to 16,733 among individuals with an average of 16,335.8 detected per library (77.8% of the gene set, Table S1). Using the same cutoff value (normalized mapped reads ≥3.0 in at least one individual), the number of combined expressed genes per treatment group ranged from 17,612 to 17,995 (average 17,840). Distribution of these expressed genes by treatment group (unique and shared) are illustrated in Figure S1. A total of 16,976 genes (90.7%) was co-expressed among all groups with similar distribution of co-expressed and uniquely expressed gene counts.

Overall variation among the treatment groups is visualized in the principal component analysis (PCA) of normalized read counts (Figure S2). Greatest separation between the DT groups was observed along PC axis 1 whereas EW samples were distinguished on axis 2. One individual from the EW control group (EW10) was uniquely distributed within the variant space. Examination of the mapped reads for this sample found four genes with skewed expression including *CD74* (CD74 molecule, major histocompatibility complex, class II invariant chain), *EEF1A1* (eukaryotic translation elongation factor 1 alpha 1), *IGLL1* (immunoglobulin lambda-like polypeptide 1), and *MARCO* (macrophage receptor with collagenous structure). Each of these are among the most highly observed loci (Table S2). With the exception of *IGLL1*, expression of these genes was significantly lower in EW10 (1.37× to 3.0×) compared to the other group samples. Expression of *IGLL1* was 4.3× to 9.8× higher in EW10.

### 2.2. Differential Gene Expression

#### 2.2.1. AFB_1_ Treatment Effects

**Figure 1:**
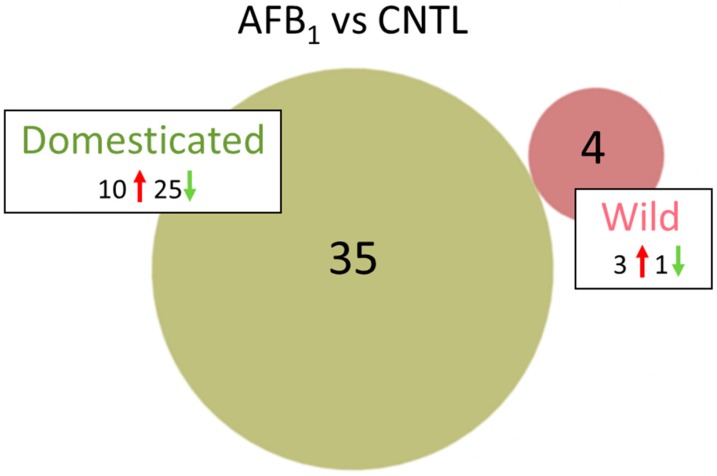
Distribution of DEGs in the turkey spleens. For each genetic type, the number of DEGs (FDR *p* < 0.05 and |log_2_FC| > 2.0) shared or unique to each treatment (AFB_1_ or control) are indicated in the Venn diagram and direction of expression change (↑ or ↓) is given for each group. Circle size is proportional to the number of DEGs.

The effects of AFB_1_ treatment on the spleen transcriptomes were considerably more prominent in the DT birds than EW. A total of 4 differentially expressed genes (DEGs) was observed in AFB_1_-treated EW compared to control birds (Table 1 and Table S3, Figure 1). Three DEGs (*KCTD16*, potassium channel tetramerization domain containing 16 [*LOC100550279*]), fatty acid-binding protein, adipocyte-like (*FABP4*); and *IL13RA2*, interleukin 13 receptor, alpha 2) had elevated expression in the AFB_1_-fed group. The fourth, *LOC104910166* a non-coding RNA (ncRNA) was down-regulated with AFB_1_ treatment. This uncharacterized locus has been withdrawn by NCBI because the model on which it was based was not predicted in the most recent turkey genome annotation (v102). Greatest fold change (FC) was seen for (*KCTD16*, log_2_FC = 5.89). The protein encoded by this gene is an auxiliary subunit that modulates receptor response of the inhibitory GABA-B receptors. Both IL13RA2, and FABP4 are implicated in inflammatory response. IL12RA2 is related to IL13RA1, a subunit of the interleukin 13 receptor complex that binds IL13 with high affinity [27]. In mammals, *FABP4* is expressed in adipocytes and macrophages and has been shown to be associated with insulin resistance, atherosclerosis and metaflammation [28].

In contrast to the EW comparison, 2353 loci were significant differentially expressed (DE) in the AFB_1_-treated DT birds compared to control-fed birds (Table 1). Of these, 242 had |log_2_FC| > 1.0 and 35 had |log_2_FC| > 2.0 with none of these genes shared with the EW AFB/CNTL comparison (Table S4). Ten of the 35 genes showed significantly higher expression in the AFB_1_-treated group and 25 were comparatively down-regulated. Greatest increase in expression was seen for *LOC100539136* (growth regulating estrogen receptor binding 1, *GREB1*), *LOC104915640* (uncharacterized protein *KIAA1755* homolog), *LOC104911153* (uncharacterized ncRNA) and *A1CF* (*APOBEC1* complementation factor) with log_2_FC > 3.0 (Table S2). Greatest down-regulation in AFB_1_-treated compared to control-fed birds was seen for *LOC104912410* (uncharacterized ncRNA), *TMSB4X* (thymosin beta 4), *LOC104910496* (amphiphysin-like, *AMPH*-like), and *LOC104915519* (myelin-oligodendrocyte glycoprotein-like).

Gene ontology (GO) enrichment analysis of the 2253 significant DE genes (Table S5) found enrichment for genes associated with core processes of gene expression with highest enrichment for the biological process categories of ribosomal small subunit assembly (GO:0000028, 9.54× enrichment, *p* = 0.0135), proteasomal ubiquitin-independent protein catabolic process (GO:0010499, 7.35× enrichment, *p* = 0.027), and cytoplasmic translation (GO:0002181, 6.82× enrichment, *p* = 2.0 × 10^–7^). Highest enrichment in GO cellular component category was proteasome core complex, alpha-subunit complex (GO:0019773, 11.7× enrichment, *p* = 0.356), cytosolic small ribosomal subunit (GO:0022627, 10.3× enrichment, *p* = 1.35 × 10^–12^) and cytosolic large ribosomal subunit (GO:0022625, 9.35× enrichment, *p* = 7.21 × 10^–16^). Pathway analysis of the DEGs in ingenuity pathway analysis (IPA) found the highest z-score (5.37, (−log(*p*) = 41.2) for Eukaryotic Initiation Factor 2 (eIF2) signaling. Studies in mammalian cells have shown that NF-κB activation and pro-inflammatory cytokine expression are dependent on the eIF2 signaling pathway [29]. Translational regulation especially of pro-inflammatory cytokines is a key innate immune response [29]. Pathway analysis also indicated activation of eIF2 signaling. eIF2 is an essential factor for protein synthesis and a critical point in stress-induced translation.

#### 2.2.2. Wild vs. Domesticated Turkey Control Groups

**Figure 2:**
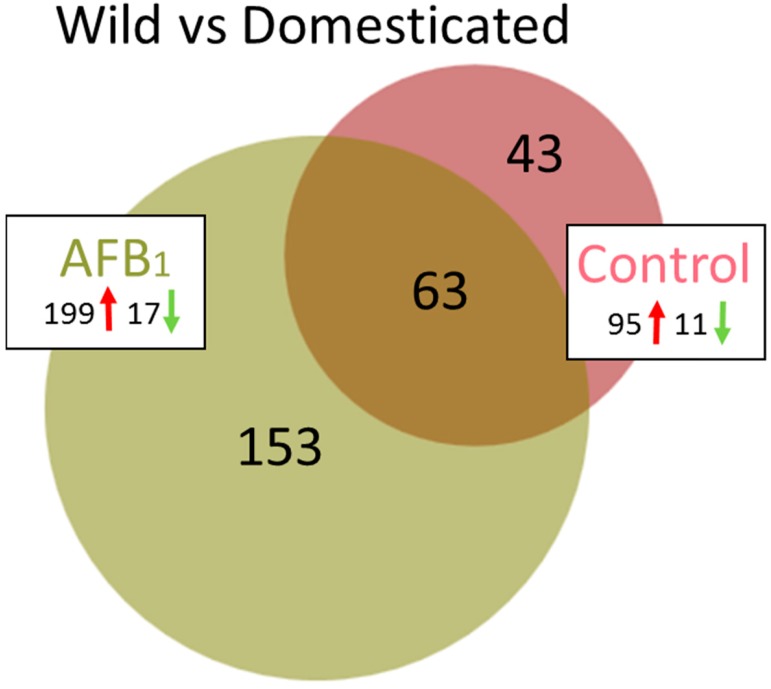
Distribution of DEGs in turkey spleens. For each comparison between turkey types (wild and domesticated), the number of shared or unique DEGs (FDR *p* < 0.05 and |log_2_FC| > 2.0) are indicated. Direction of expression change (↑ or ↓) is given for each group and circle size is proportional to the number of DEGs.

Transcriptome comparison between the control groups (EW versus DT) found 344 DEGs (false discover rate (FDR) *p* < 0.05, Table 1) with log_2_FC ranging from −6.38 to 6.60 (Table S3). A total of 106 of these DEGs had |log_2_FC| > 2.0 (Figure 2, Table S6) and the majority (95) showed higher expression in the EW group. Of the 106 genes, 63 were shared in common in the comparison with AFB_1_-treated birds (Figure 2). Analysis of the 106 DEGs in the Database for Annotation, Visualization and Integrated Discovery (DAVID) [30] found enrichment (E score = 1.96) for a group of membrane-associated genes *AQP8* (aquaporin 8), *CLRN3* (clarin 3), *DIO2* (deiodinase, iodothyronine type II), *FER1L6* (fer-1 like family member 6), *LOC100548708* (epithelial chloride channel protein-like), *TMEM27* (transmembrane protein 27) and solute carrier family genes (*SLC1A1*, *SLC5A1*, *SLC5A8*, *SLC7A14*, *SLC7A9*, *SLC22A4* and *LOC100543156* [solute carrier family 22 member 13-like]). Cluster analysis identified two significant annotation clusters including transporter activity (GO:0005215, *p* = 0.00074), and integral component of membrane (GO:0016021, *p* = 0.0082). In addition, pathway analysis in IPA identified higher expression of enzymes important in hormone metabolism and degradation such as sulfotransferases (6B1-like, *LOC104912373*; 1C1-like, *LOC100545251*) and glucuronosyltransferase (UDP-glucuronosyltransferase 1-1-like, *LOC100547885*).

Of the 43 DEGs unique to the control group comparison, greatest up-regulation in the EW birds was seen for *BRINP3* (bone morphogenetic protein/retinoic acid inducible neural-specific 3, log_2_FC = 6.6) and *LOC104915513* (histone deacetylase 7-like, log_2_FC = 6.2). In mammals, BRINP3 is primarily expressed in the brain, and has been shown to be targeted to mitochondria and to inhibit neuronal cell proliferation [31]. Histone deacetylase 7 is important in FOXP3 transcriptional regulation, cell cycle progression and development [32]. Up-regulation of transcriptional regulators such as histone deacetylase 7 (*LOC104915513*) potentially influences downstream transcriptional regulators such as FOXP3, important in the development and inhibitory function of regulatory T-cells [33]. Greatest down-regulation in the EW group was seen for 14-3-3 protein gamma-B (*LOC104917314* [*YWHAG*], log_2_FC = –6.4). This gene is part of a family of adapter proteins associated with the regulation of several signaling pathways, apoptosis, cell cycle, and stress response [34,35].

#### 2.2.3. Wild vs. Domesticated Turkey AFB_1_-treated

**Figure 3:**
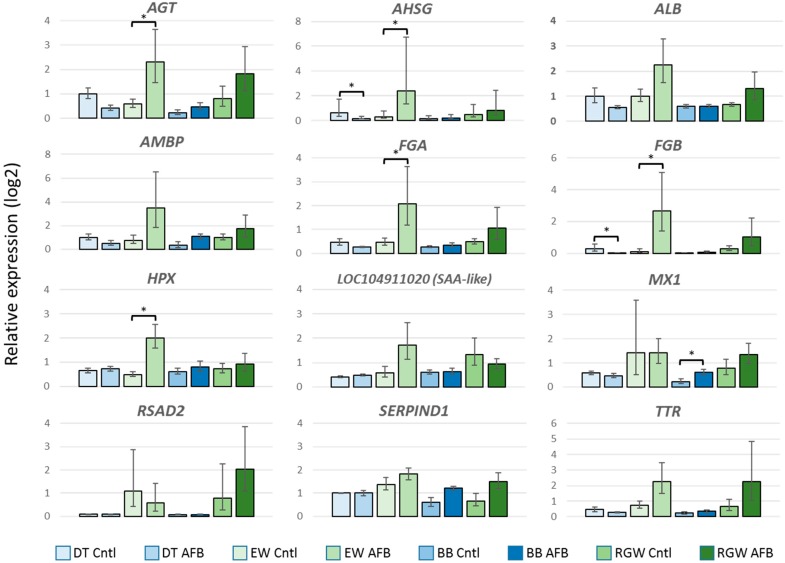
Effect of AFB_1_ on relative gene expression (log_2_) of selected genes in the spleens of turkeys as measured by quantitative real time PCR (qRT-PCR). For each gene, the fold change (ΔΔCt) between AFB_1_-treated and control birds is given. Bars denote 1 SE of the mean. DT = domesticated, EW = Eastern wild, BB = Broad Breasted White domesticated, and RGW = Rio Grande Wild. * significant comparisons (*p* < 0.05).

Compared to the control groups, a greater number of genes showed significant expression differences between the EW and DT birds following AFB_1_ treatment. A total of 435 DEGs (FDR *p* < 0.05) were observed with 216 having |log_2_FC| > 2.0 (Table 1). Expression of these 216 DEGs was directionally biased, with 199 showing greater expression in EW birds and only 17 with higher expression in the DT group (Figure 2, Table S7). As discussed above, 63 DEGs were shared with the control comparison. Among the unique DE genes highest up, regulation was observed for *LOC104915725* (mitochondrial ribosome-associated GTPase 1-like), *AVPR1A* (arginine vasopressin receptor 1A), *SLCO4C1* (solute carrier organic anion transporter family, member 4C1), and *LOC100541395* (thyroid hormone-inducible hepatic protein-like), each with log_2_FC > 5.0 (Table S7). Largest down regulation was observed for *LOC104915640* (uncharacterized protein KIAA1755 homolog), *LOC104917003* (zonadhesin-like), *LOC104916581* (7-dehydrocholesterol reductase-like), and *LOC104917133* (uncharacterized ncRNA), all with log_2_FC < –4.0. Only one of the unique down-regulated genes (*OPTC*, opticin) was also DE in the control group comparison. Interestingly, expression of the primary (*CYP1A5*) and secondary (*CYP3A37*) hepatic AFB_1_-activating P450s was significantly higher in the DT birds. Two of the shared genes that showed the highest expression differences were the INF-inducible genes *MX1* (MX dynamin-like GTPase) and *RSAD2* (radical S-adenosyl methionine domain containing 2). Both of these genes are believed to function in antiviral immune response. Further examination of these genes by quantitative real time PCR (qRT-PCR) supported elevated expression in EW birds in both control and AFB_1_-treatment groups. We also examined other domesticated (Broad Breasted White, BB) and wild birds (Rio Grande Wild, RGW, *M. g. intermedia*) to further contrast the genetic types. Both of these groups of birds (BB and RGW) showed similar expression patterns as the DT and EW comparison with higher relative expression observed in the RGW birds compared to BB (Figure 3). Interestingly, wild birds of both subspecies (EW and RGW) generally displayed higher levels of inter-individual variation in the genes assayed.

**Figure 4:**
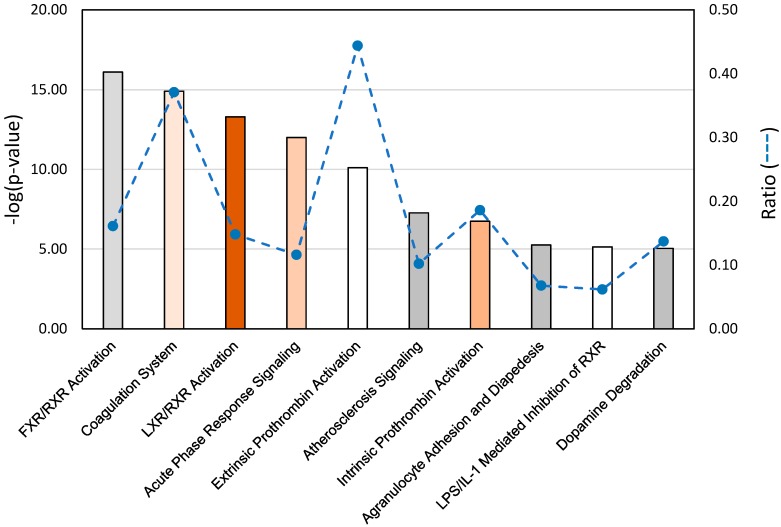
Significant gene pathway associations identified in ingenuity pathway analysis (IPA) of spleen RNAseq data from AFB_1_-treated Eastern Wild turkeys compared to domesticated turkeys. *p* values are assigned to canonical pathways based on differential expression (DE). Bar plot provides the 10 (statistically significant threshold = 1.3) pathways with the largest ratio scores (dashed line). Color of bars indicate predicted activation or inhibition (z-score) of the pathway. White bars are those with z-score approximately equal to 0, orange bars have positive z-scores and gray bars indicate pathways with no prediction. Intensity of color indicates magnitude of z-score.

Gene pathway analysis of the DEGs identified in the EW vs. DT comparison revealed highest significance for the canonical pathways representing FXR/RXR and LXR/RXR activation, coagulation system, acute phase response signaling, prothrombin activation (extrinsic and intrinsic), and atherosclerosis signaling (Figure 4). These pathways are necessarily related as 27 of the DE genes in these pathways co-occur in at least one other pathway, and some genes like *SERPINA1* (LOC100542070, alpha-1-antitripsin) and *FGA* (fibrinogen alpha chain), occur in six and five of the pathways, respectively. Within these pathways, affected genes were primarily up regulated in EW compared to DT as would be expected given the overall abundance of up-regulated genes in this comparison (92%). Expression differences are particularly evident in the IPA acute phase response signaling pathway with significantly higher expression observed in EW birds for several genes (Figure S3). Given the extra-hepatic expression of these genes, 10 (*AGT*, *AHSG*, *ALB*, *AMBP*, *FGA*, *FGB*, *HPX*, *SAA*-like [*LOC104911020*], *SERPIND1*, and *TTR*) were chosen for further examination and confirmation by qRT-PCR. Elevated expression in each of the 10 genes in EW birds was supported compared to DT birds (Figure 3). All showed greater expression in EW birds treated with AFB_1_ compared to controls and compared to AFB_1_-treated DT birds indicating up-regulation in response to AFB_1_. Expression of seven genes was reduced by AFB_1_ treatment in DT birds suggesting the opposite effect. For other domesticated (Broad Breasted White, BB) and wild birds (Rio Grande Wild, RGW), gene expression as measured by qRT-PCR supported the findings seen in qRT of the EW and DT birds.

## 3. Discussion

The combination of high toxicity and ubiquitous presence in feed, makes AFB_1_ a serious concern for the poultry industry. Consumption of AFB_1_ has widespread physiological effects that adversely affect poultry production. Exposed birds display general poor performance, decreased growth and reproductive depression [3,36]. Consistent with immunotoxicity, AFB_1_ also impairs cell-mediated, humoral, and phagocytic functions [2,13,14,15]. In addition, AFB_1_ exposure results in compromised immune response, making birds more susceptible to infectious diseases [15]. Importantly, immunotoxic affects and blood-clotting abnormalities may occur at AFB_1_ doses lower than required to elicit reduced performance [36,37]. The present study quantifies gene expression within the turkey spleen transcriptome and identifies key expression differences between genetically susceptible and resistant birds.

The effect of AFB_1_ on the spleen is the combined result of the systemic toxin and damage signaling from the liver. Exposure to AFB_1_ in poultry is known to deplete splenic lymphocytes and generate circulating lymphocytopenia [3,38,39]. Pathomorphological studies have shown significant reduction in the density of lymphoid cells through lymphocytic degeneration [40,41,42]. Pathological changes in the spleen are thought to result from increased oxidative stress, decreased glutathione (GSH) levels and increased apoptosis [17,43]. Studies suggest that effects on the spleen are dependent on the effective exposure and genetic susceptibility of the birds. For example, Zhu et al. [43] reported significantly lower relative spleen weights in broiler chickens fed AFB_1_-contaminated diets (0.8 mg AFB_1_/kg feed), whereas Peng et al. [44] reported a significant increase (82–134 µg AFB_1_/kg). Grozeva et al. [42] found significantly lower relative spleen weights in AFB_1_-fed commercial strain turkey poults (0.2 and 0.4 mg AFB_1_/kg), whereas Quist et al. [25] reported slightly lower (non-significant) spleen weight in AFB_1_-fed wild turkeys (100–400 µg AFB_1_/kg). To facilitate rapid collection of tissues, splenic weights were not recorded in the present study, however, the effects of AFB_1_ on body weight and liver mass in these same birds are summarized in a companion study of hepatic gene expression [12].

The spleen is unique in its combination of discrete functions enabling innate and adaptive immune responses [45]. Up-regulation of a diverse set of coagulation factors, cell cycle regulators, and Nrf2-mediated response genes was previously observed in turkey embryos with AFB_1_ exposure [46]. Our comparison of AFB_1_-treated EW vs. DT birds identified significant association of DEGs with the canonical pathways for coagulation system and acute phase response signaling. Coagulation activation produces proteases that can interact with cell receptors and induce signaling pathways to up-regulate pro-inflammatory mediators [47] thereby increasing inflammation. Gene expression changes in the coagulation system are consistent with altered coagulation times observed in poultry following AFB_1_ exposure [25,37,48].

Acute-phase proteins (APPs) are proteins that respond to infection or tissue damage (inflammation) either by a significant increase (positive) or decrease (negative) in plasma concentration [49]. As a component of the innate immune system, APPs have antimicrobial, coagulatory, negative feedback functions [50]. APPs and their role in the acute phase response are widely reported in both humans and other mammals (reviewed in [51]). Although primarily synthesized by hepatocytes in the liver, expression of APPs is common in other tissues, even under normal conditions [52]. In this regard, focal expression of APPs in the turkey spleen in response to AFB_1_ is not unexpected. In the chicken, five APPs, including alpha 1-acid glycoprotein (*AGP*), serum amyloid A (*SAA*), *PIT54* (turpentine-induced 18-B), C-reactive protein (*CRP*), and ovotrasferrin (*OVT*), are expressed in extrahepatic tissues of healthy birds [53]. In particular, *SAA* was highly expressed in the secondary lymphatic tissues of the chicken cecal tonsil and spleen. As seen in the RNAseq and qRT analyses, several APPs were expressed at significantly higher levels in wild turkey spleens compared to those of domesticated birds in response to AFB_1_ treatment. Many of these same genes were also similarly expressed at higher levels in the livers of these same birds [12].

Our study of the effects of AFB_1_ on splenic gene expression in domestic turkeys [23] found that acute AFB_1_ exposure suppressed innate immune transcripts, especially from antimicrobial genes. Also evident was up-regulation of transcripts indicative of either increased cytotoxic potential or activation-induced cell death in the spleen during aflatoxicosis. This earlier study was conducted prior to the availability of the turkey whole-genome sequence and relied on de novo assembly of RNAseq reads to identify gene transcripts. Comparison of AFB_1_-treated DT birds with controls found 391 transcripts to be differentially expressed. Although the majority (88.4%) of the significant differentially expressed transcripts unique to the AFB_1_-treatment group were up-regulated, transcripts from several innate immune genes, such as lysozyme G (*LYG*), leukocyte cell-derived chemotaxin 2 (*LECT2*), beta-defensin 1 (*THP1*), and beta-defensin 2 (*THP2*) were significantly down-regulated. Given the limitations in equating gene IDs in the current genome annotation to transcripts in the Monson et al. study [23], comparison of DEGs in the present study identified only 22 (representing 20 genes) common to both studies. The majority of these had small relative expression changes (|log_2_FC|< 2). For example, in the present study expression of *LECT2* and *THP2* were slightly lower in EW birds treated with AFB_1_ compared to DT birds but did not show a significant response to AFB_1_ compared to EW controls. However, other genes like UMP-CMP kinase 2 mitochondrial (*CMPK2*) were uniquely upregulated in EW. This gene may function in terminal differentiation of monocytic cells and as an enzyme in the nucleotide synthesis salvage pathway [54].

Domesticated turkeys, like humans, lack hepatic alpha-class GSTAs with high activity toward AFB_1_ [55]. In contrast, livers of wild and heritage turkeys possess GST-mediated AFBO detoxification activity [10] and are relatively resistant to aflatoxicosis [25]; suggesting a loss-of-function concomitant with genetic selection for the modern commercial bird. The effects of AFB_1_ in domesticated turkeys and the lessened effect in wild birds is multifactorial. Although the hepatic transcriptome is dysregulated by AFB_1_ in all turkeys, significant differences between wild and domestic birds are apparent in the expression of phase I and phase II drug metabolism genes, and genes involved in cellular regulation, modulation of apoptosis and inflammatory responses [12,46]. Further evidence for the role of genetic background in response to AFB_1_ is seen in the differential activation of the inflammatory response in the spleens of wild birds compared to their domesticated counterparts consistent with our studies of the cecal tonsil (intestine) of the same birds [26]. Greater inter-individual variation in gene expression as measured by qRT-PCR was also observed in the wild birds. Combined, these studies demonstrate that in addition to the presence of functional hepatic GST-mediated AFB_1_ detoxifying capability, wild turkeys possess a capacity to more effectively respond to AFB_1_ exposure.

## 4. Materials and Methods

This study used turkey strains (Eastern wild (EW, resistant) and domesticated (DT, susceptible)) previously demonstrated to vary in AFB_1_-detoxifying GST activity. Animal husbandry and the AFB_1_ treatment protocol were as described in Reed et al. [12]. All procedures were approved by Utah State University’s Animal Use and Care Committee (Approval #2670, 26 September 2016).

### 4.1. RNA Isolation, Sequencing and RNAseq Data Analyses

Total RNA isolation, quantification and preparation for sequencing was as previously described [12]. RNA Integrity Numbers (RIN) averaged 6.2 and libraries (four replicate samples per treatment group) were prepared and sequenced (101 bp paired-end reads) at the University of Minnesota Genomics Center (UMGC) on the HiSeq 2000 using v2 chemistry (Illumina, Inc.). Raw data were deposited in the NCBI’s Gene Expression Omnibus (GEO) repository as SRA BioProject 346253. Analysis of RNAseq data followed the protocols outlined in Reed et al. [12].

### 4.2. Quantitative Real-Time PCR

Quantitative real-time PCR (qRT-PCR) of AFB_1_-treated and control animals was used to test expression of select genes. Selected for analysis were the wild and domesticated turkey used for RNAseq analysis, plus other samples of genetically distinct domesticated (Broad Breasted White, BB) and wild birds (Rio Grande subspecies, RGW, *M. g. intermedia*) from a parallel challenge experiment. Of the six samples from the DT and EW groups used for qRT-PCR, four were in common with the RNAseq study. Synthesis of cDNA was performed on DNase-treated mRNA with Invitrogen Super Script IV First-strand synthesis kit (Invitrogen, Carlsbad, CA, USA). PCR primers were designed with Primer3 software [56]. Primer sets were designed using the turkey genome (UMD5.0) to span at least one intron (exon/exon junction) to limit artifact DNA amplification. Normalizing genes were tested for uniformity and the most stable reference (ribosomal protein L4, *RPL4*) was determined with RefFinder [57]. Stability values for *RPL4* were 0.209 and 0.266 as calculated by Normfinder and Genorm, respectively. Quantitative analysis of gene-specific amplicons was done using the Quanta PerfecTa Sybr Fastmix (Quanta, Biosciences, Inc, Gaithersburg, MD, USA) run on a CFX96 touch real-time detection system (BioRad, Hercules, CA, USA). Each target gene reaction was run in triplicate, with duplicate normalization, no template and gDNA control reactions. Expression was normalized to *RPL4* and interpreted by Double Delta Ct Analysis (ΔΔCt, [58]) using the standard workflow within the CFX Maestro software package (1.0, Bio-Rad, Hercules, CA, USA).

## Figures and Tables

**Table 1:** Summary of gene expression in pair-wise comparisons of treatment groups in the aflatoxin B_1_ (AFB_1_) study.

Comparison	Groups	Total Expressed Genes	Shared Genes	Unique Genes in Each Group	FDR *p* < 0.05	|log_2_FC| > 1.0	|log_2_FC| > 2.0	Up/Down Regulated
AFB_1_	EW (AFB vs. CNTL)	18,417	17,536	422/459	4	4	4	3/1
	DT (AFB vs. CNTL)	18,155	17,254	543/358	2353	242	35	10/25
Genetic Line	CNTL (EW vs. DT)	18,307	17,300	695/312	344	223	106	95/11
	AFB (EW vs. DT)	18,372	17,383	575/414	435	351	216	199/17

For each comparison, the treatment groups, total number of expressed, shared and unique genes, are given. For genes with significant false discovery rate (FDR) *p* values, the number of differentially expressed genes (DEGs) with either |log_2_(fold change (FC))| > 1.0 and > 2.0 are shown. The number of up and down regulated genes are given for DEGs with |log_2_FC| > 2.0. Genes were considered expressed in a treatment group if the by-total normalized read count was ≥ 3.0 in any individual within the group.

## Supplementary Materials

The following are available online at https://www.mdpi.com/2072-6651/11/5/242/s1, Figure S1: Distribution of expressed genes in turkey spleen by treatment group; Figure S2: Summary of principal component analysis (PCA) of by-total normalized RNAseq read counts; Figure S3: Effect of AFB_1_ on expression of turkey genes in the IPA canonical pathway “acute phase signaling”; Table S1: Summary of RNAseq data for turkey spleen transcriptomes; Table S2: Mean quality-trimmed RNAseq read counts for turkey spleen from two turkey types (wild and domesticated); Table S3: Summary of pair-wise differential gene expression analysis of spleen transcriptomes; Table S4: Genes showing significant differential expression in AFB_1_-treated versus control groups in domesticated turkey; Table S5: Summary of overrepresentation test (PANTHER (GO Consortium release 20150430, [59]) of the 242 differentially expressed genes (|log_2_FC| > 1.0) in the AFB_1_-treated DT birds as compared to controls; Table S6: Genes showing significant differential expression (FDR *p* < 0.05 and |log_2_FC| > 2.0) in EW versus DT control groups; Table S7: Genes showing significant differential expression (FDR *p* < 0.05 and |log_2_FC| > 2.0) in comparison of EW versus DT AFB_1_ groups.

## Abbreviations

AFB_1_	aflatoxin B_1_
AFBO	exo-AFB1-8,9-epoxide
APP	acute phase protein
BB	Broad Breasted White
Ct	threshold cycle
CYP	cytochrome P450
DE	differentially expressed
DEG	differentially expressed gene
DT	domesticated turkey
EW	Eastern wild turkey (*Meleagris gallopavo silvestris*)
FC	fold change
FDR	false discovery rate
GO	gene ontology
GST	glutathione S-transferase
IPA	Ingenuity Pathway Analysis
ncRNA	non-coding RNA
PCA	principal component analysis
qRT-PCR	quantitative real-time polymerase chain reaction
RGW	Rio Grande wild turkey (*Meleagris gallopavo intermedia*)
