# Supplementary material for: Altered Gene Response to Aflatoxin B1 in the Spleens of Susceptible and Resistant Turkeys
Source: Toxins (Basel). 2019 Apr 28;11(5):242. doi: 10.3390/toxins11050242 (PMC6562755; doi:10.3390/toxins11050242)

Figure S1: Distribution of expressed genes in turkey spleen by treatment group. Number and percent of genes are included. Genes were considered expressed in a treatment group if the by-total normalized read count ≥ 3.0 in any individual within the group. EW CNTL = Eastern wild control, DT CNTL = domesticated control, EW AFB = Eastern wild aflatoxin B_1-treatment and DT AFB = domesticated aflatoxin B_1-treatment.

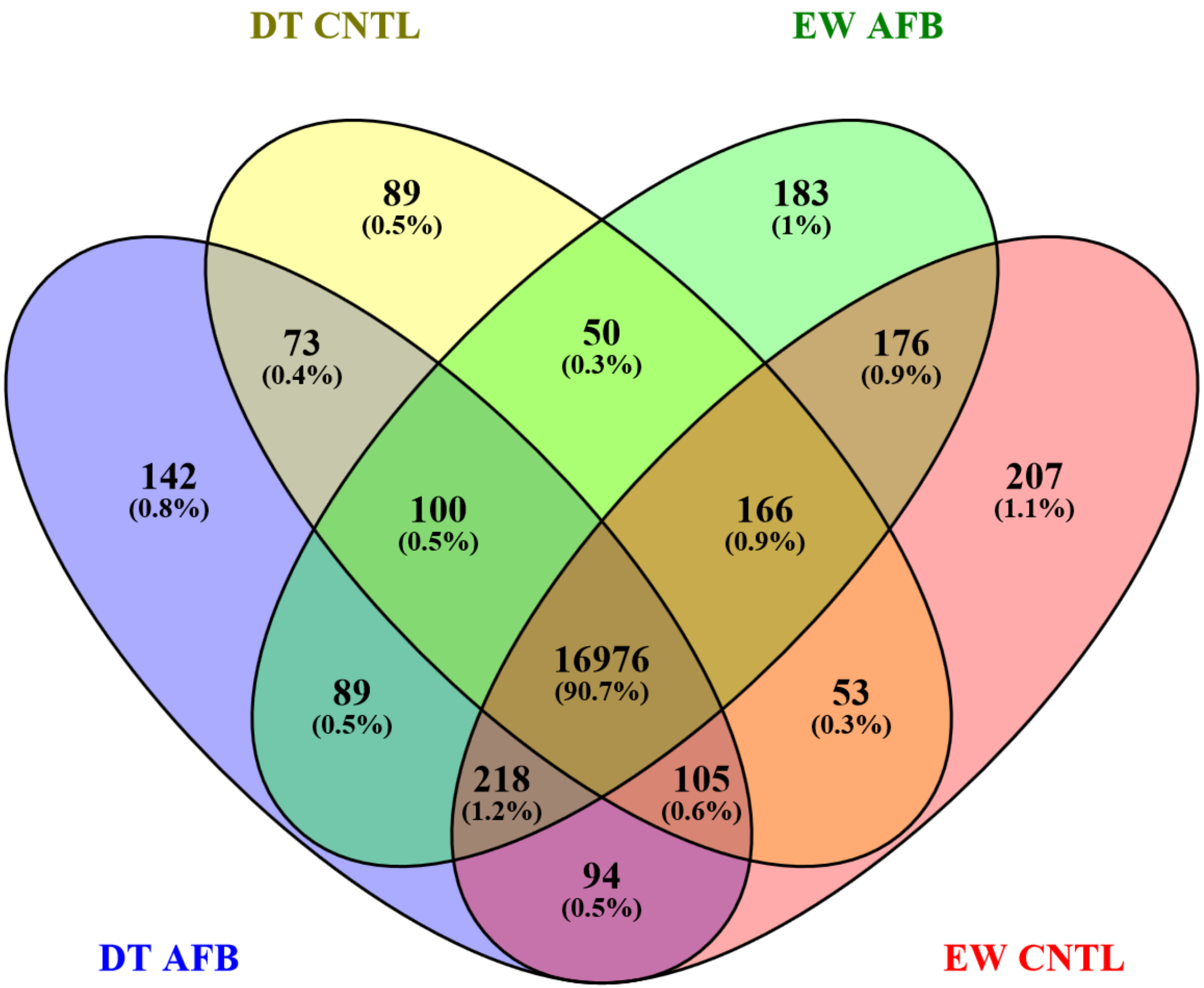

Figure S2: Summary of principal component analysis (PCA) of by-total normalized RNAseq read counts. Sample to sample distances are illustrated on the first two principal components.

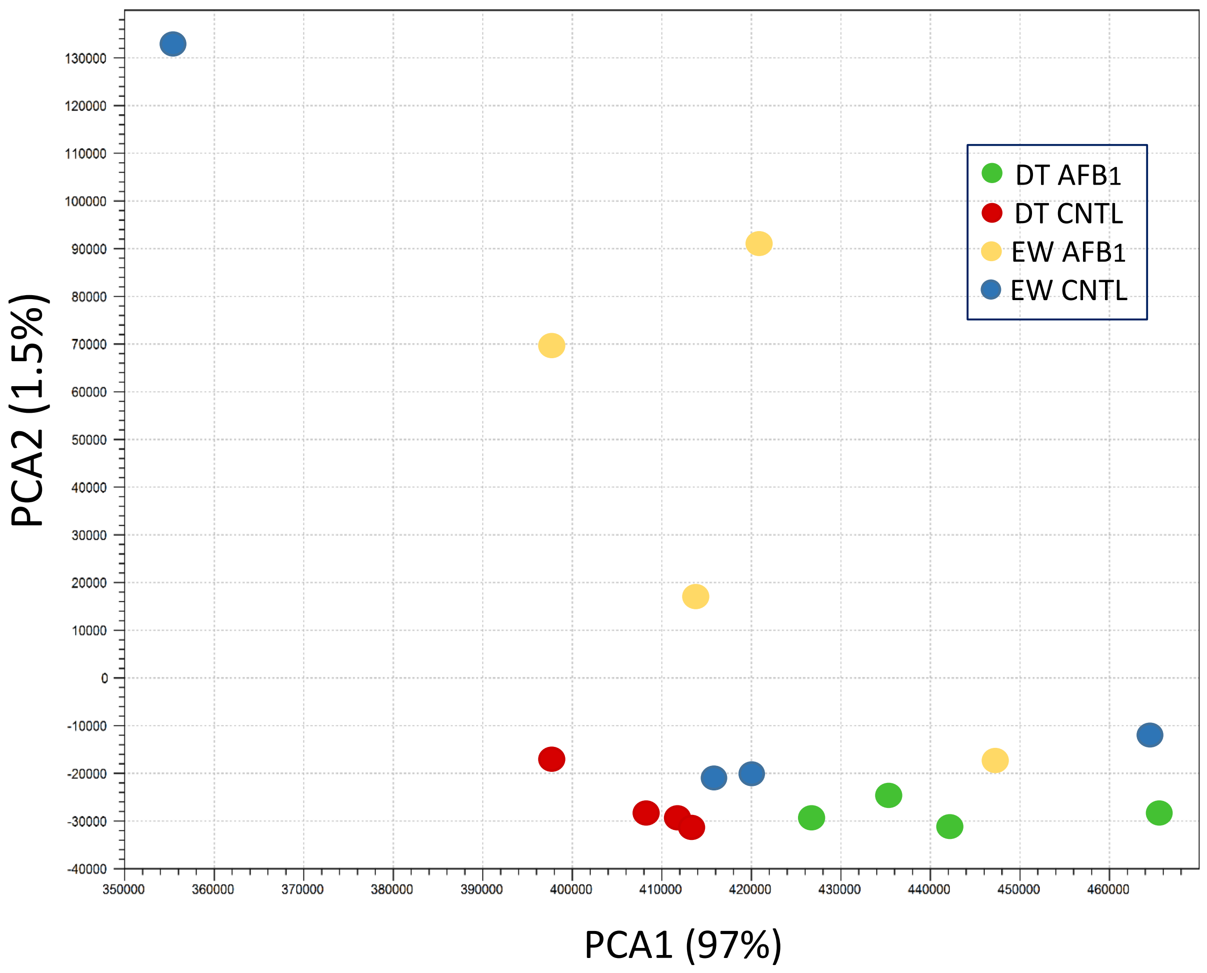

Figure S3: Effect of AFB_1 on expression of turkey genes in the IPA canonical pathway “acute phase signaling”. Genes with significantly higher expression in EW birds compared to DT following AFB_1 treatment are indicated in red.

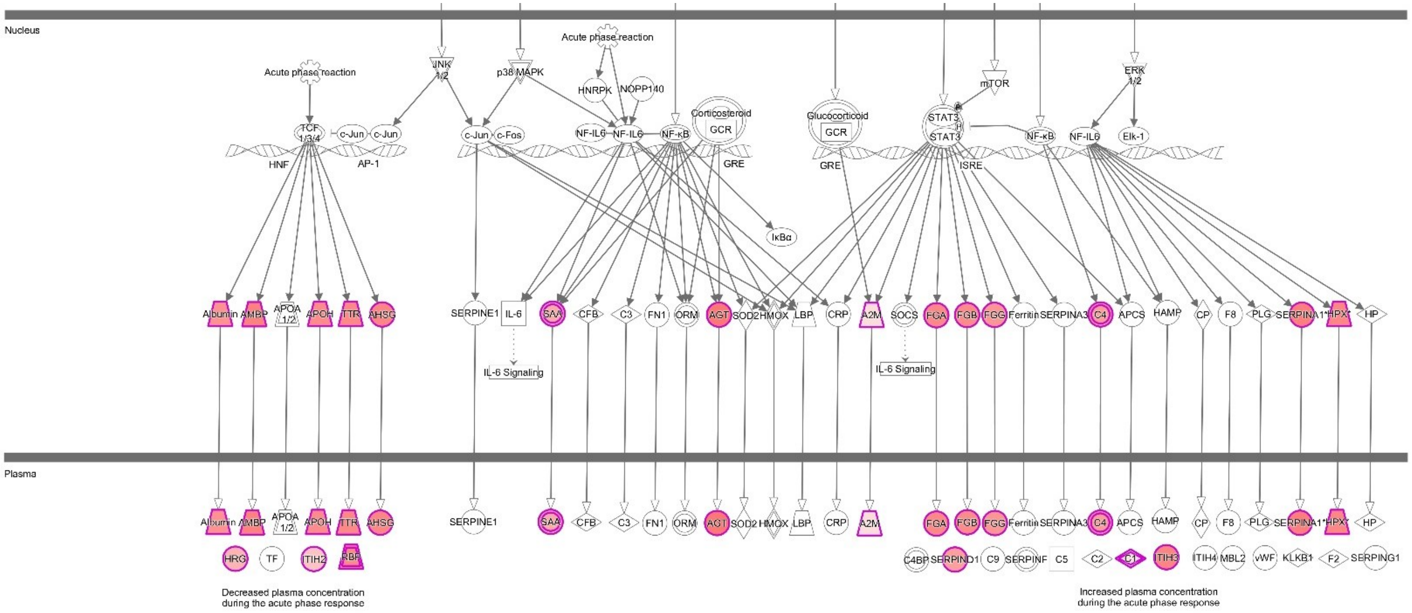

Supplement: Supplementary file 1 [file toxins-11-00242-s001.zip › Supplementary Figures and Tables/Supl Figures.pdf]
